# E-waste it wisely: lessons from Africa

**DOI:** 10.1007/s42452-022-04962-9

**Published:** 2022-02-05

**Authors:** Thomas Maes, Fiona Preston-Whyte

**Affiliations:** GRID-Arendal, Teaterplassen 3, 4836 Arendal, Norway

**Keywords:** E-waste, Africa, EEE, WEEE, Basel Convention, Bamako Convention

## Abstract

E-waste is the world’s fastest growing and most valuable domestic waste stream. The increasing production of e-waste is driving elevated levels of export from developed to developing countries. Although countries worldwide are actively recognising the issues around e-waste and introducing policies, legislation or regulations governing e-waste, a large fraction of e-waste, goes undocumented at its end-of-life. Much of the global e-waste is accumulating in open dumpsites in several African countries. Using available data, we calculate the total e-waste in Africa (locally produced plus imported e-waste) for 2019 to be between 5.8 and 3.4 metric tonnes (Mt). This is believed to be an underestimate, large data gaps exist, hindering more precise estimates. The data is further complicated by, sometimes intentional, differences in labelling and reporting between formal and intermittent informal importers. Based on the available data, the main African recipients of e-waste are Nigeria, Ghana, and Tanzania, with Kenya, Senegal and Egypt featuring as countries of concern. The lack of proper waste management in the recipient developing countries, leads to environmental contamination and human exposure. A coordinated, regional and global, approach is needed in tackling e-waste. Regulatory frameworks, together with monitoring and compliance mechanisms need to be developed, financed, and enforced.

## Introduction

The increase in mismanaged waste has been reported around the world, made visible by plastic pollution. Global waste production continues to grow with increasing population and increasing per capita consumption associated with economic growth [1]. With increasing economic and technological development, dependence and rapid obsolescence of electronics increased [2, 3]. This makes e-waste the world’s fastest-growing domestic waste stream [4]. Global volumes have doubled in the last decade [5]. Containing rare-earth and other metals, it is also one of the most valuable waste streams per volume. Overall, the value of selected raw materials contained in e-waste in 2019 was equal to $57 billion USD, corresponding to a total of 25 Mt [4]. In addition to existing predicted increases of e-waste [6] and despite the economic impact of the pandemic, global e-waste may increase rapidly during and post COVID-19, driven by demands for electronics due to remote working and home schooling [7].

### E-waste: definition and types

**Fig. 1:**
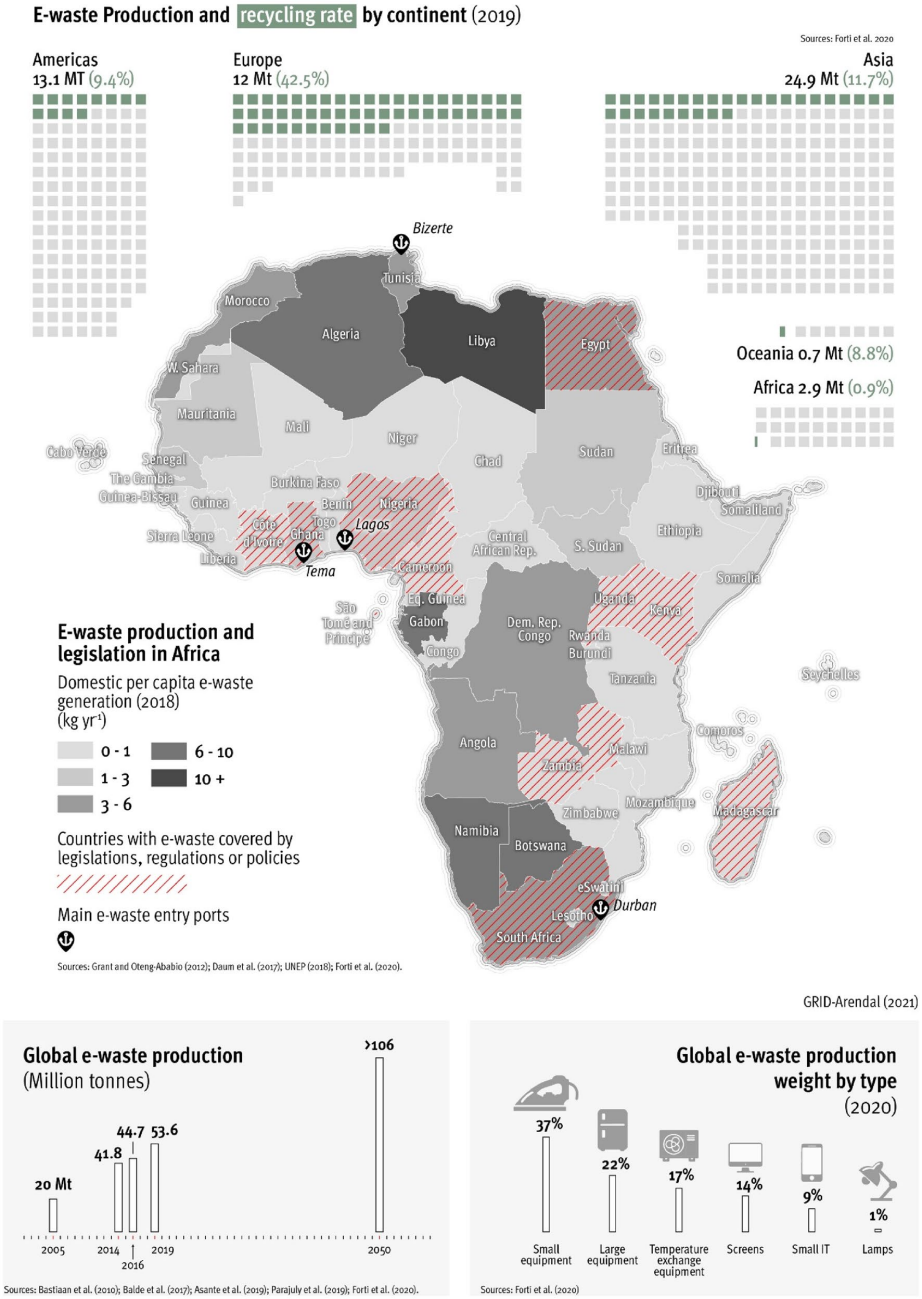
The African e-waste perspective

Though legal definitions are still being agreed, E-waste, is a term used to cover all waste items of electrical and electronic equipment (EEE) and their parts (Fig. 1) [8]. Items which are intentionally discarded, without the intent of reuse, are also called Waste EEE (WEEE). Used EEE on the other hand is second hand—meant to be functional but includes non-functional, but repairable items.

### E-waste: global production of e-waste

In 2016, 8.9 Mt of e-waste was documented to be collected and recycled globally, corresponding to 20% of e-waste generated that year (44.7 Mt) [9]. This decreased to 17.4% of 53.6 Mt in 2019. Over 5 years (2014–2019), e-Waste volumes grew by 21%, and the 2014 volumes are predicted to double by 2030 [4]. The discrepancies in volumes produced and percentage properly collected and recycled (Fig. 1), indicates that a large fraction goes undocumented and is untreated and/or illegally transported and leaking [4].

### E-waste: regulatory frameworks and legislative drivers

Countries worldwide are actively recognising the issues around e-waste and introducing domestic policies, legislation or regulations governing e-waste. In 2014, just 44% of the world’s inhabitants were protected by legal waste related frameworks, this increased to 66% in 2017 and 71% with 78 countries by October 2019 [4]. In Africa, the two conventions regulating e-waste are the Basel Convention and Bamako Convention.

Global regulatory frameworks such as the Basel Convention (1992) directly deals with e-waste as a hazardous waste. The Basel Convention on the “Control of Transboundary Movements of Hazardous Wastes and Their Disposal” is a multilateral treaty focussed on reducing environmental and human health risks resulting from hazardous waste trading. In 2019, changes were made to Annexes II, VIII and IX of the Basel Convention (during the 14th Conference of the Parties). These amendments aim to improve the control of hazardous plastic waste movements across country boundaries. Amongst others, it requires written notification and approval for such movement across boundaries. Exemptions are made for EEE intended for reuse, thus reducing hazardous waste formation and extending the life of EEE. However, discussions within the Basel Convention on the differentiation between waste or “intended for reuse” are ongoing [4].

To help achieve the objectives of the Basel Convention (article 11), the convention encourages parties to organise regional, multilateral and/or bilateral agreements on hazardous waste, which—resulted in the Bamako Convention (1998). This is a treaty consisting of African countries forbidding the import of any hazardous waste, with the aim of reducing and managing e-waste transport nationally and across African states. In theory, this ensures disposal of electronics in accordance with clean and green environmental principles [3].

Though not always effective, legislation exists to prevent hazardous waste trade in countries exporting waste to developing countries. Challenges arise due to slow regulatory progress and low levels of legislative enforcement [4]. In Europe, this issue is regulated by the Waste Shipment Regulation (2006), which includes provisions to address uncontrolled and illegal waste exports, and the EU Waste Framework Directive (2008). The last one was recently revised to modernise and streamline its provisions, to focus on inspections and includes waste management principles such as the “polluter pays principle” or the “waste hierarchy”.

### E-waste end-of-life options and leakages

Monitoring global quantity and streams of e-waste is difficult, no harmonised end-of-life measurement methods exist for all countries [10]. A large fraction of e-waste, 82.6% or 44.3 Mt in 2019, goes undocumented at its end-of-life [4]. The increasing production of e-waste is driving elevated levels of export from developed to developing countries [11]. Owing to the lack of appropriate waste management in the recipient developing countries, including open burning of residual unrepairable fractions, this leads to local contamination, human exposure, and crop contamination [11]. The majority of these exports are illegal or disguised as being intended for reuse or as scrap or even worse, described as charitable donations [3]. Though measuring the exact volume of the global flow of e-waste is difficult, it is widely accepted that the volume is significant, with transboundary movement of e-waste and used EEE accounting for 7–20% of e-waste generated [4]. A large part of the global e-waste is accumulating in open dumpsites in several African counties, notably Ghana and Nigeria [2].

To quantify “leakage”—a term used for illegally exporting waste, the Basel Secretariat receives member states reports on illegal waste trades. From 2015 to 2017, the Basel Action Network (BAN) put 314 trackers on electronic waste items sent to recycling facilities across ten European Union (EU) countries. The data indicated that 6% of traced items (19) were exported from country of origin. Over half of those items (11) were destined for developing countries, making these exports “highly likely to be illegal” [12]. The other 8 exported trackers were found in other EU member states. The non-EU destination countries included Nigeria, Ghana, Tanzania, Hong Kong, Pakistan, and Thailand. In total 64% of the discovered exports, that left the EU, were taken to Africa (7 items). The United Kingdom (UK) was the country responsible for shipping the most electronics outside the EU to less developed nations, with 5 tracked items. BAN [12] estimates that a total of 352,474 Mt of e-waste is exported from EU countries to developing nations every year. It is likely that the exports to Africa have increased as a result of the Chinese National Sword Policy, 2017. The transboundary European export rate (6%) falls far below the United States of America (USA) export rate of 40% [12]. The USA is the only industrialised country not to ratify the Basel convention nor its amendments and has insufficient laws controlling e-waste exports [3].

## Transboundary movement of e-waste to Africa

### Estimated volumes and hotspots

Even though monitoring the transboundary movement of e-waste into Africa is notoriously difficult, three African countries, two on the West coast, Ghana, and Nigeria, and one on the East coast, Tanzania, have been identified as recipients of e-waste from the EU/UK [11–13]. Other recipient countries within Africa have also received e-waste from developed countries [5, 11, 14]. Import data for e-waste is currently lacking for the Americas and Asia, however, one can assume many other port cities in Africa are similarly receiving large volumes of the same types of discarded European consumer products [12] as well as from the USA and other developed nations [15]. Two West African countries, Ghana and Nigeria have high direct imports of e-waste, with Nigeria the leading importer of used EEE on the African continent [11, 14]. Approximately 60,000–71,000 t of used EEE were imported annually into Nigeria through the two main ports in Lagos in 2015 and 2016 [16]*.* In Nigeria, 77% of used EEE imported during this period originated from the EU [9, 11]*.* Additionally, three of Africa’s most active ports: Durban (South Africa), Bizerte (Tunisia), and Lagos (Nigeria) have all been identified as the major ports of entry of used EEE to the African continent [17]. This indicates that, shipments containing hazardous materials still circumvent the Basel and Bamako Conventions by passing through busy environments, illegally, through methods such as mislabelling [18].

### Production of e-waste in Africa

Globally, per-capita e-waste generation rates vary geographically [11]. The e-waste generation in Africa, with an average annual per capita e-waste generation of 2.5 kg, is well below Europe (16.2 kg) and the Americas (13.3 kg) [4, 9]*.* Africa locally generates between 50 and 85% of its total e-waste, the remainder originating from illegal transboundary imports from developed countries in the Americas, Europe, and China [15]. About 2.9 Mt of e-waste was generated in Africa in 2019, with the highest e-waste generating countries, also being major population centres [19], identified as Egypt, South Africa and Nigeria [4]. Though considering kg per capita per year generation, Libya (10 + kg capita^−1^ year^−1^) is the highest per capita generator, followed by Algeria, Botswana, Gabon, Namibia (6–10 kg capita^−1^ year^−1^) [11]. Using the above figures from SBC [15] and Forti et al. [4], we calculate the total e-waste in Africa (locally produced plus imported e-waste) for 2019 to be between 5.8 Mt and 3.4 Mt. Based on the data gaps presented by others and in this review, we consider this to be an underestimation.

### Quality of imports into Africa

In addition to the locally generated waste, Africa must process imported transboundary e-waste. This importation of e-waste can have the unintended consequence of displacing these developing economies’ capacity to recycle their own domestic waste [20]. A lot of this e-waste is introduced as “charitable donations” and “second-hand goods” [3, 18], the implication being that these goods are functional. Workshop data from Accra, Ghana in 2019, showed that out of 0.215 Mt of e-waste imported: 30% was not waste, but “new” products (unused stocks), 14% was second-hand—possible to repair or keep using, leaving 56% as actual waste needing processing [21]. The exact numbers vary between studies [15, 16, 22], but all show e-waste (30%, 15% and 19% respectively) imported into Ghana as usable EEE. The data is further complicated by differences between formal and intermittent informal importers, with Grant and Oteng-Ababio [17] reporting a decrease in working condition from 70 to 60% between shipment types into Nigeria.

### End-of-life options

Globally, EEE end-of-life options include reuse, repair, refurbishing, repurposing of parts into other products, recycling and resource recovery, landfill (both controlled disposal and sanitary engineered landfilling), incineration uncontrolled and indiscriminate dumping and littering. Within Africa, most of these options are utilised—dictated by infrastructure, markets, and value items.

With built in obsolescence [2, 3], reuse, repair and refurbishing of used EEE is not the primary option globally. The lifetime of used EEE imported into Africa is often extended through informal repair. However, with many imported used EEE close to their end of life, the shortened lifespan eventually increases the amount of e-waste within Africa [23]. For products that cannot be fixed, disassembly occurs. Leading to the reuse of parts in other, different, EEE or goods [2].

Recycling rates of e-waste vary across the continent and are difficult to compare due to different interpretations. South Africa recycles 9.7% of its generated e-waste, in that it is dismantled and the valuable fractions exported for recycling (beneficiation) [24]. While Ghana rather processes 40–60% of its domestically generated e-waste of which 95% is done by the informal sector [23]. The difference between both being that the informal sector typically doesn’t “recycle”—they collect for recycling, they repair for reuse, but they don’t beneficiate which would require expensive pyrometallurgical or hydrometallurgical facilities. Recycling complexity varies across the continent, with South Africa, Egypt, Morocco, Namibia, and Rwanda having some formal recycling e-waste industry (supported by the informal industry) [4, 24, 25]. While the industry in Ghana and Nigeria is solely driven by the informal sector. In long term treatment sites, such as Agbogbloshie (Ghana), informal recycling of e-waste has been vertically integrated into a functional e-waste economy [26–29].

Formal industrial incineration of waste does not occur in Africa, with the exception of one facility, Reppie WtE, in Ethiopia. Most recognised landfills are uncontrolled, nor sanitary engineered [11]. Resource recovery, or “urban mining” is widespread in the informal handling of e-waste in Africa [11], although it is alleviating the depletion of natural resources, the unregulated manner in which it takes place is an extremely polluting process [5]. The e-waste which is not reabsorbed into the markets through the above processes accumulates in illegal dumpsites, is openly burnt, or littered in the African environment.

## E-waste impact in Africa

### Environmental impacts

**Fig. 2:**
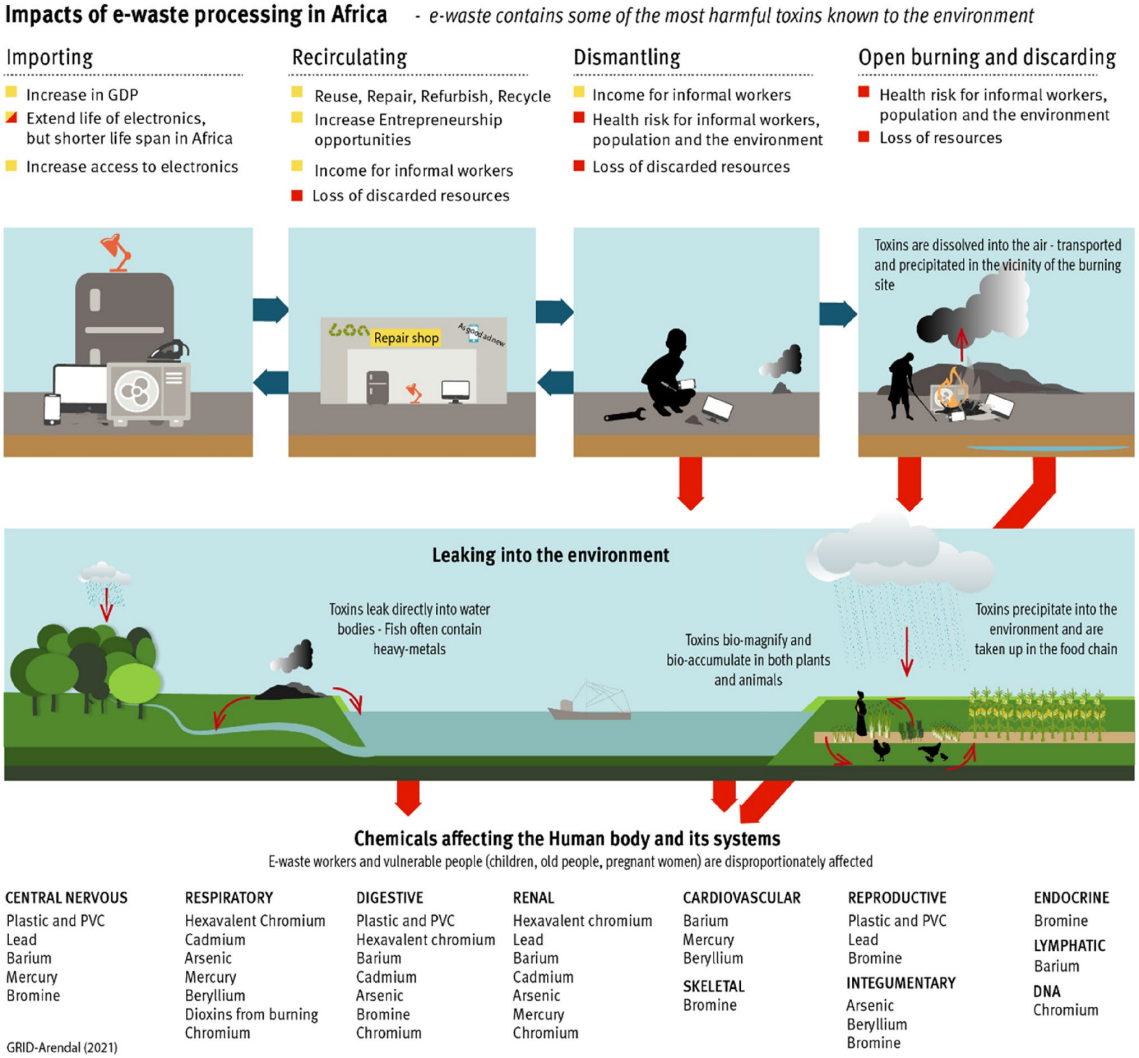
Impacts of e-waste processing in Africa

The toxic and cancerous pollutants released into the environment during improper treatment of e-waste at end-of-life (Fig. 2), include heavy metals such as lead, cadmium, and mercury, as well as dioxins, furans, and polycyclic aromatic hydrocarbons [12]. Within Africa, close to e-waste processing sites, toxic elements, persistent organic pollutants (POPs), and heavy metals have been observed in elevated levels in dust, soils [30–33] and vegetation, including edible plants [34, 35]. Further environmental effects have been observed because of higher metal and rare earth element (REEs) [36] concentrations in downstream aquatic and marine environments, causing adverse marine consequences including smaller, sicker, and sparser fish stocks [37, 38]. REEs are contaminants of emerging concern [36]. Heavy metals, organic pollutants, and higher concentrations of Polychlorinated Biphenyls (PCBs) [39] have been found in aquatic environments, as well as polycyclic aromatic hydrocarbons (PAHs), oxygenated PAHs, trace metals and metalloids in fish samples [37]. These compounds can be linked to e-waste processing [39, 40] and are toxic to several aquatic species [40].

On average, electronics consist for 17% of plastics, mostly high-quality recyclable polymers like HDPE, although they often contain hazardous compounds, e.g. flame retardants, which make them difficult to recycle. In South Africa remanufacturing of WEEE plastics is mostly (80%—7500 t) exported [41], this is likely to be similar in other African countries. The remaining plastic fractions are in the best case landfilled, but more likely to be unrecovered, dispersed into the environment in a range of sizes due to fragmentation resulting from informal recycling processes. Furthermore, depending on landfill characteristics, recent surveys have indicated that larger plastics fragment and landfill leachates may act as another source of microplastics. Microplastics discharged from these sites may pose greater risks to human and environmental health by sorbed toxic and persistent hazardous chemicals [42].

Current e-waste processing in Africa has the potential to impact the ozone layer and climate change. Open burning, not only releases toxins but also carbon dioxide (CO_2_). The importation of old cooling devices, such as refrigerators or air-conditioning units’, and the improper and unsafe disposal of these devices’ leads to the release of ozone depleting substances (ODS) and greenhouse gasses (GHG) [4, 43]. Additionally, the inefficiency of these items during use undermines Africa’s climate efforts through energy inefficiency [44]. In contrast, “urban mining” contributes to an uncaptured amount of metal resource recovery.

### Human health

The rudimentary processing of the e-waste in informal sites through manual removal, open burning and open acid digestion leads to widespread contamination [5]. The toxins released into the environment bioaccumulate in human tissues [44–48], and have been found, along with PAHs [46, 49, 50] in e-waste workers [46–48, 51] and surrounding populations [29, 52]. These toxins lead to several adverse health effects (Fig. 2), including carcinogenic, non-carcinogenic [53], mutagenic [54, 55], genotoxic [35], neurotoxicity and endocrine disruption [56] and neonatal issues [3]. Further health issues, specifically to e-waste workers, include physical injuries, chest and respiratory tract associated symptoms, malaria, headaches, body pains and stomach discomfort [51].

### Economic impacts and societal impacts

The transport of used EEE and e-waste to Africa, has resulted in an informal, yet important industry, with far reaching consequences. Used EEE, and repairable EEE, have permitted individuals and companies to buy inexpensive and vital electronics or IT equipment [2], helping socioeconomic development [11, 57]. Informal e-waste recycling provides a major source of livelihood for many poor urban communities [29]. The informal sector contributes to 25% of the e-waste recycling in South Africa, with an estimated workforce of 10,000 with 2000 regular workers in 2013 [58]. Within Ghana, in 2014, e-waste activities generated US$105–268 million, creating employment for at least 200,000 people nationwide [23]. Agbogbloshie, described as the biggest e-waste site in sub-Saharan Africa [51], creates 4000—6000 direct jobs [59], supporting strong entrepreneurship and economic opportunities through the development of community-based collection, recovery, and recycling businesses [2].

E-waste in Africa is ambivalent in its nature. The rudimentary processing of the informal sector creates elevated risk for health and environment alike [5], affecting the entire surrounding population and ecosystem. Such pollution and health effects will have long term environmental, social and economic consequences. Inversely, e-waste processing allows for entrepreneurship, job creation, reuse, and refurbishment for sale of cheap electronics, less waste and recapturing of metals [2]. Globally, as Extended Producer Responsibility (EPR) schemes develop, the cost of end-of-life and recycling will become built into the cost of EEE. This increase in costs will have societal effects, increasing the technological gaps already present in our world [3]. Bridging these gaps is too often used to justify the environmental racism present in the current transboundary movement of e-waste [60].

## Discussion

### Future projections

Prior to 2012, Nigeria and China received the majority of the global cross-border transport of e-waste [35], this changed in 2011 when China introduced the Regulation on Management of the Recycling and Disposal of Waste Electrical and Electronic Equipment [61]. Over the past decade, illegal e-waste transport to African countries, such as Egypt, Ghana, Kenya, Nigeria, Senegal and Tanzania, received attention in the international news [11]. The growing global manufacture of electronics, in combination with the absence of action plans in African countries and the lack of infrastructure for proper and sustainable e-waste management [2], has led to e-waste becoming a growing African problem, with global origins. Global forecasted scenarios for e-waste generation show an increase of more than 100% in the next 30 years [6]. The use of EEE in Africa is low but growing at a staggering pace [11, 15], for example, the e-waste in Senegal, Uganda and South Africa is projected to increase by a factor of two to eight in the next 10 years [62], and e-waste inflows into Ghana were projected to double in three years between 2017 and 2020 [22]*,* however no new data is available to verify these projections.

### Lessons learned and further considerations

Transboundary movement of e-waste is dynamic, reacting to social, economic, and regulatory changes [4]*.* A coordinated, regional and global, approach is needed in tackling e-waste. Given the flow of e-waste to Africa, the Basel Convention is lacking in practical enforcement. Given the flow of e-waste within the continent, highlighted by the import of e-waste to Agbogbloshie, Ghana through South Africa, Tunisia, and Nigeria [18], enforcement of the Bamako Convention can also be improved*.*

E-waste, when treated as a waste to be discarded leads to the loss of valuable metals and REEs contained within. Modest recycling rates of metals combined with rising demand for high-tech goods containing them, require increased mining with its associated social, environmental, health, energy, water, and carbon-footprint costs [63]. Mining and metallurgical processes produce significant amounts of waste and constitute one of the biggest challenges to the environment [64]. Additionally, a positive correlation between mining and conflict on a local level has been detected—the historical rise in mineral prices might explain up to 1/4 of the average level of violence across conflict African countries over the same period. The effect of mining on communities, notably its catalytic and shifting effects on relocation and urban development, are important drivers of urbanisation in Africa [65].

Rigorous international environmental directives, including UNEP goals aimed at improved recycling of WEEE [66], the restriction of the use of hazardous substances in EEE [67], and EPR [68] programmes all have made recycling an important responsibility. Environmental pressures and climate change are leading to companies and supply chains considering new models for environmental protection. The “Circular Economy” (CE) model was established as a sustainable approach, able of dissociating economic development from material utilization and waste production [69]. Greener options aim to apply WEEE waste to turn it into simple, available and viable polymetallic secondary REE supplies. To secure a stable and more environmentally friendly supply, attention has turned towards “urban mining”. Consistent with the CE approaches of the 4R scheme (Reduce, Reuse, Remanufacture and Recycle), the “urban mining” method offers several advantages, leading to improved energy efficiency and lower demand for mining of new raw materials [70], and so lowering the mining footprint. In a near future, by unlocking the potential that WEEE provides as a material, proper and efficient WEEE recycling will become a very important sector from economic and environmental perspectives.

The absence of e-waste infrastructure and e-waste legislation are some of the challenges facing e-waste management in Africa [11, 71]. Africa is the worst covered continent regarding e-waste legislation [5]. However, the legal landscape of Africa is changing rapidly, with the number of African countries covered by e-waste legislation, policy or regulation rising from 3 in 2018 [11] to 13 in 2020 [4]. Subregional approaches are also occurring within the East Africa community, adopting a regional e-waste strategy [72], and an enforcement programme to monitor and control transboundary movements of used EEE customised for Benin, Egypt, Ghana, Nigeria, and Tunisia. A scheme for exchanging information on used EEE between exporting and importing states was also developed [11, 14]*.*

Policy or regulatory frameworks need reachable targets and effective enforcement [4]. For enforcement, timely response and finance is key [3]. Enforcement of conventions lacks funding in Africa. Given the rising environmental and health cost of e-waste in Africa, this commodity should become an enforcement priority. Waste related funding should be orientated towards incentive programmes constructing recycling infrastructure and in country technology across Africa. Currently, CE in the WEEE industry has been addressed mainly in Europe and Asia [69]. In the absence of a full CE, there are few options for financing WEEE management globally and in Africa. Funding of convention implementation for the processing of e-waste comes in four forms: public financing, private financing, public–private partnerships, and donors and grants.

Public financing is a common practice in Asia, this involves the funding of WEEE management services by the government through budget provision [73]. This practice proves difficult in Africa. Existing reasons for the poor management of waste in Africa, include, amongst others, feeble organisational structures; lack of suitable skills; insufficient funds; weak regulation; lack of enforcement; low public awareness; corruption, conflict; political instability; and absence of political will [74].

Private financing involves private sector funding of WEEE management services and running of the provision formally or informally. In Africa, the informal e-waste sector dominates the entire WEEE chain from collection to extraction. Regardless of the creations in WEEE “recycling” businesses, a major gap is that tackling the issue demands integrated multi-actor interventions with multiple stakeholders to reduce WEEE imports, while improving safe and efficient recycling capacity.

Public–private partnerships (PPP) merge the government and private sector in supplying resources and management skills for WEEE services [75]. Public–private partnerships with results-based-financing offers opportunities for WEEE management in Africa as it allows for innovation and locally appropriate solutions while focusing on agreed WEEE targets.

The last option is to rely on the use of donors and grants. This can be combined with a PPP to cater for a specific WEEE management aspect. Several international conventions or agreements recognise, the need for a financial mechanism to fund and assist developing country Parties with respect to both mitigation and adaptation in continuation of their existing obligations under these conventions. The potential of WEEE recycling in terms of a country's greenhouse gas (GHG) reduction target would offer a new path towards climate-change mitigation [76]. The call for financial assistance from Parties with more financial resources to those that are less endowed and more vulnerable by the United Nations Framework Convention on Climate Change (UNFCCC), the Kyoto Protocol and the Paris Agreement might be useful in the financing of WEEE management. The Global Environment Facility (GEF) funds are available to developing countries and transition economies to achieve the goals of the international environmental conventions and agreements. GEF assistance is given to government agencies, civil society organizations, private sector companies, research institutions, among the broad diversity of potential partners, to carry out projects and programs in recipient countries.

It is important to note that African countries have varying GDP’s, population figures, centres and social systems, and associated income and cost streams. As such, no single financial model will be suitable for all countries, but rather a mixture of the above. The informal sector is important in e-waste management in Africa. The informal nature of the e-waste processing industry ensures that the social injustices faced by the workers and surrounding marginalised communities [77], go beyond the issue of e-waste [77]. As the most vulnerable workers, often migrant [3, 77, 78], gravitate to the work. To prevent the industry moving further into the shadows, legal development and enforcement needs to take a supportive approach [51]. Countries should engage with informal waste collectors, protecting the informal workers with adequate personal protection and making sure e-waste is collected and sent to licenced recyclers for efficient and safe processing [79].

Sustainable funding such as through EPR schemes, supportive legislation, stakeholder partnerships, and inclusion of the existing informal and formal sectors are needed to increase the recycling capacity and decrease the amount of WEEE contaminating the environment and endangering human health. Charges for EEE sold by producers should be added by classifying EEE according to their eventual end-of-life treatment requirements and cost. As such improved design would be financially rewarded, allocate costs of historic waste proportionately (on the basis of tonnes of new products sold), and provide sufficient financial guarantees against future waste costs and liabilities [80]. Furthermore, in line with the Paris Agreement, as part of a global effort, developed country Parties should “continue to take the lead in mobilizing climate finance from a wide variety of sources, instruments and channels, noting the significant role of public funds, through a variety of actions, including supporting country-driven strategies, and taking into account the needs and priorities of developing country Parties” (UNFCCC 2015).

### Recommendations

To achieve a comprehensive implementation regime globally and regionally, a rigorous enforcement of the Basel [5] and Bamako Conventions is needed while encouraging outlying states to ratify the conventions. However, this would not solve the current loopholes, nor the lack of enforcement allowing for the illegal transboundary movement [3, 11, 15]. Regulatory frameworks, together with monitoring and compliance mechanisms need to be developed and financed.

E-waste processing in Africa is driven by poverty, unemployment, and socioeconomic needs in Africa [11], as well as public and private sector design globally. The e-waste treatment technologies available globally are not necessarily appropriate, nor economically viable in Africa. Valuable and appropriate innovations, appropriate for Africa are arising within Africa—and African centric solutions should be supported and developed. There is a need to create efficient, effective, and clean “urban mining” systems in Africa [11], combined with an aim to recuperate materials from e-waste, processing them within Africa (so that resources, especially metals, are not exported out of Africa for value increased processing elsewhere) thus, preserving resources, controlling pollution while creating jobs and wealth for Africa.

Africa is set to undergo a major social and economic transformation over the next century as its population explodes, cities urbanise and consumer purchasing habits change [11]. This will lead to an increase in locally generated e-waste—as well as the increasing global e-waste and associated imports. Creating robust data on generation and movement of e-waste will help both the private and public sector in planning, financing, and developing healthy e-waste processing that combines the existing informal industry and their expertise into the formal green economy. Proper assessments on pilot projects are needed and this knowledge needs to be shared [3]. Developing the policies and legal frameworks now, for collection and processing, considering planning and financial instruments, will be hugely beneficial for the African public and private sectors to design e-waste processing in Africa that meets environmental standards and supports a thriving industry.

Globally, change is needed too. Countries exporting used EEE to Africa (or elsewhere) need to ensure that their second-hand exports are working prior to export or can be easily fixed and are not damaged in transport. In other words, that the used EEE is not e-waste. Additionally, design change to facilitate repairability, reuse and recycling of e-waste is an imperative, including but not limited too; modularity, reduction, and ultimate removal of substances of very high concern (SVHC), clear ingredients, minimum standards for recycled content. Lastly, EPR plays an important part in both creating a circular economy product and financing collection and end of life options.
